# Creating the Geriapolis – How a Small Award Can Build a City

**DOI:** 10.1093/geroni/igaf122.1360

**Published:** 2025-12-31

**Authors:** Benjamin Rosenstein

**Affiliations:** University of Minnesota, Minneapolis, Minnesota, United States

## Abstract

The University of Minnesota Medical School has no geriatrics focused training such as rotations, clinics, or a fellowship program. Additionally, we have limited geriatrics teaching faculty. The time and money necessary to develop geriatrics education in medicine, and collaborations with interdisciplinary health professions schools, does not exist. Unless it is mandated by a grant. My GACA is aimed at overcoming the long-standing barriers to furthering geriatrics education and care at our institution. The GACA provides me with the time and finances necessary to develop and design new curriculum and establish collaborations. I aim to develop geriatrics education vertically (from medical school to practice) and horizontally (across health professions), creating a foundation for ongoing geriatrics education where it had not previously existed. This presentation will highlight the challenges I faced in promoting geriatrics education and how the GACA has provided me with support to achieve success. I will provide examples by reviewing current projects including establishing dementia and delirium education, collaboration with our longitudinal rural care program, and the wide-spread networking needed to develop a long-term care medical student experience. I will then focus on questions that may arise with future plans such as establishing an interprofessional geriatrics clinic, creating inpatient geriatrics experiences, and using the ECHO platform to maximize educational reach.

